# Cellular Metabolism: A Fundamental Component of Degeneration in the Nervous System

**DOI:** 10.3390/biom13050816

**Published:** 2023-05-11

**Authors:** Kenneth Maiese

**Affiliations:** Cellular and Molecular Signaling, New York, NY 10022, USA; wntin75@yahoo.com

**Keywords:** Alzheimer’s disease, apolipoprotein E (APOE-ε4), AMP activated protein kinase (AMPK), autophagy, dementia, diabetes mellitus, erythropoietin, COVID-19, mechanistic target of rapamycin (mTOR), pyroptosis

## Abstract

It is estimated that, at minimum, 500 million individuals suffer from cellular metabolic dysfunction, such as diabetes mellitus (DM), throughout the world. Even more concerning is the knowledge that metabolic disease is intimately tied to neurodegenerative disorders, affecting both the central and peripheral nervous systems as well as leading to dementia, the seventh leading cause of death. New and innovative therapeutic strategies that address cellular metabolism, apoptosis, autophagy, and pyroptosis, the mechanistic target of rapamycin (mTOR), AMP activated protein kinase (AMPK), growth factor signaling with erythropoietin (EPO), and risk factors such as the apolipoprotein E (APOE-ε4) gene and coronavirus disease 2019 (COVID-19) can offer valuable insights for the clinical care and treatment of neurodegenerative disorders impacted by cellular metabolic disease. Critical insight into and modulation of these complex pathways are required since mTOR signaling pathways, such as AMPK activation, can improve memory retention in Alzheimer’s disease (AD) and DM, promote healthy aging, facilitate clearance of β-amyloid (Aß) and tau in the brain, and control inflammation, but also may lead to cognitive loss and long-COVID syndrome through mechanisms that can include oxidative stress, mitochondrial dysfunction, cytokine release, and APOE-ε4 if pathways such as autophagy and other mechanisms of programmed cell death are left unchecked.

## 1. Introduction and Overview

Metabolic disorders can affect all systems of the body. In particular, they can be a significant contributor to cognitive loss and dementia and impact both the central nervous system and the peripheral nervous system. Present therapies that may involve pharmacological treatments, immunomodulators, and lifestyle changes for metabolic and neurodegenerative disorders can only address, under most conditions, symptomatic relief and cannot ultimately stop disease progression. Novel investigations into the cellular disease mechanisms of metabolic cellular dysfunction provide exciting and new therapeutic avenues for consideration to treat neurodegenerative disorders with the understanding that these pathways are complex and must be carefully considered to limit any undesirable clinical outcomes.

## 2. Cellular Metabolism Dysfunction

Cellular metabolism dysfunction, which includes diabetes mellitus (DM), has been rising in prevalence more quickly in low- and middle-income countries than in high-income countries (Table 1). Almost eighty percent of adults with DM are living in low- and middle-income countries [1]. The prevalence of DM has increased from nine and one-half percent during the period of 1999 to 2002 to twelve percent during the period of 2013 to 2016. A number of parameters can affect disease prevalence, such as education, co-morbidities, and socioeconomic status [2,3,4,5]. Almost thirteen percent of adults with less than a high school education have DM compared to ten percent of individuals with a high school education and DM. If one has greater than a high school education, the risk decreases to seven and one-half percent. Additional risk factors for developing complications of DM consist of tobacco consumption, hypertension, exercise, elevated serum cholesterol, and obesity [6,7,8,9,10,11,12,13]. In regard to obesity, increased body weight leads to impaired glucose tolerance that results in DM progression [14,15,16,17,18,19,20,21,22,23,24]. Obesity can especially increase the risk of developing DM in young individuals and can affect stem cell proliferation, aging, inflammation, oxidative stress injury, and mitochondrial function [25,26,27,28,29,30,31,32,33,34].

At least 500 million individuals are believed to suffer from DM throughout the world [2,6,8,35,36,37,38]. Interestingly, approximately forty-five percent of the four million annual deaths that occur in individuals with DM affect those under the age of seventy [39]. In the United States (USA), thirty-five million individuals, approximately ten percent of the population, are diagnosed with DM [40,41]. In addition, greater than four hundred million individuals are believed to either suffer from metabolic disease or be at risk for developing DM [1,14,16,22,26,42,43,44,45,46,47]. Many of these individuals have not received a diagnosis of DM, and it is estimated that at least seven million individuals over the age of 18 are undiagnosed with DM. As an example, it was estimated that almost thirty-five percent of adults in the USA has prediabetes based on their fasting glucose and hemoglobin A1c (HbA_1c_) levels [3,48].

The financial expenditures for the care and treatment of individuals with DM are considered to be significant [2,37,39,47,49]. At minimum, USD 20,000 per year is required to care for each individual with DM. The care for patients with DM can now exceed USD 760 billion [1]. Treatment and care for individuals with DM uses more than seventeen percent of the gross domestic product in the USA per the Centers for Medicare and Medicaid Services (CMS) [50]. An additional seventy billion USD is consumed for individuals with DM as a result of disability and functional loss.

## 3. Cellular Metabolism in the Nervous System

Diseases of the nervous system are a significant component of non-communicable diseases (NCDs) [5,51,52]. At least seventy percent of the annual deaths that occur each year are the result of NCDs [53,54]. An observed rise in NCDs parallels an increase in the life expectancy of the global population [55,56,57,58]. Life expectancy is now approaching eighty years of age [59], with a one percent decrease in the age-adjusted death rate from the years 2000 through 2011 [60]. In addition, India and China, examples of developing nations that are growing, will see their elderly population increase from five to ten percent over future years [7,61]. Increased lifespan may be due to multiple factors that include improved access to healthcare, targeted sanitation measures, and greater public health measures that address populations at the highest risk of disability (Table 1). These measures can lead to earlier and more effective treatment for multiple chronic disorders [56,62,63,64,65,66,67,68,69,70,71,72].

The improvements in healthcare access and a concurrent rise in lifespan for the global population has resulted in an increased prevalence of neurodegenerative disorders [28,41,73,74,75,76,77,78]. Diseases of the nervous system represent over six hundred disorders that lead to death and disability [61,76,77,79,80]. Neurodegenerative disorders affect more than one billion individuals, approximately fifteen percent of the global population, and at least seven million individuals die each year from neurodegenerative disorders [81]. Furthermore, the cost of neurodegenerative disorders is greater than USD 700 billion in the United States (USA) alone. This includes caring for disorders such as cognitive loss, stroke, back pain, epilepsy, trauma to the nervous system, and Parkinson’s disease (PD). Of note, dementia care is the most significant cost factor with more than USD 800 billion a year allotted for cognitive care [54]. These financial projections do not account for the additional expenses necessary to provide adult living care, social outreach programs, and companion care, since almost 60 million new health and social care workers will be needed [53,54,82].

Dementia also is considered to be markedly under-diagnosed and treatment may not begin until twelve to eighteen months after a proper assessment is made [39,83,84]. If one examines the sporadic cases of Alzheimer’s disease (AD), this disorder is expected to increase over time [6,64,81,85,86,87]. Per reports from the World Health Organization [54], dementia affects all countries in the world and it is now considered to be the seventh leading cause of death [41,84,88,89,90]. The sporadic form of AD represents most of the clinical cases for this disorder. It is estimated that at least ten percent of the global population over the age of 65 is affected by sporadic AD [6,47,73,86,87,91,92,93].

**Figure 1:**
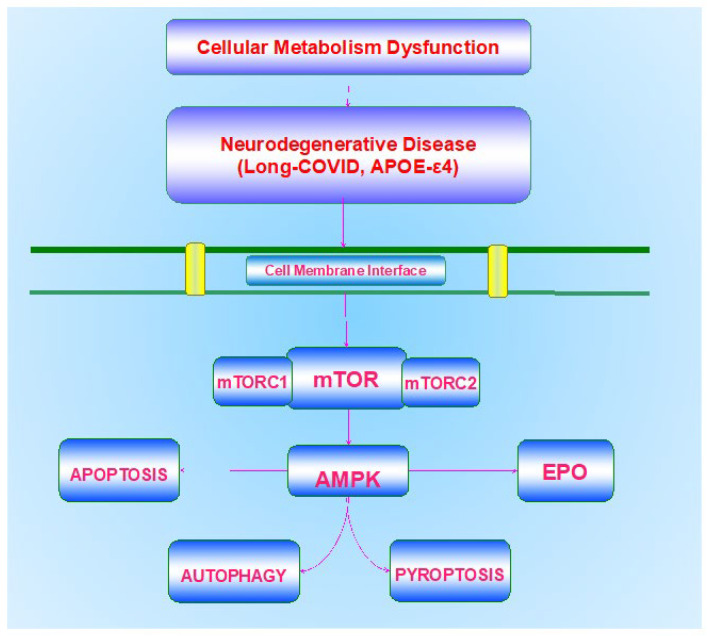
**Circadian Rhythm Pathways during Cognitive Impairment**. Therapeutic strategies that can target cellular metabolism, apoptosis, autophagy, and pyroptosis, the mechanistic target of rapamycin (mTOR), AMP activated protein kinase (AMPK), growth factor signaling with erythropoietin (EPO), and risk factors such as the apolipoprotein E (APOE-ε4) gene and coronavirus disease 2019 (COVID-19) can offer exciting potential for the treatment of metabolic disorders linked to neurodegenerative disease. Conditions that involve long-COVID syndrome can be multi-factorial and involve metabolic pathways, programmed cell death mechanisms, mTOR pathways, growth factors, and APOE-ε4.

In regard to metabolic disorders such as DM, this disorder affects all systems of the body that involve the central nervous system, the peripheral nervous system, the inflammatory system, and the vascular system (Figure 1). In the central nervous system, DM can be a significant contributor to cognitive loss and dementia [6,27,39,47,94,95,96,97,98]. DM can result in insulin resistance and dementia that occurs in patients with AD [84,98,99]. DM can affect impact stem cell proliferation [7,32,33,34,100], cytoprotective pathways [22,29,98,101,102,103], retinal disease [38,104,105,106], and immune-mediated pathways that involve microglia [95,106,107,108,109,110,111]. Furthermore, over seventy percent of diabetic individuals can develop peripheral neuropathy. DM can lead to both autonomic neuropathy [112] and peripheral nerve disorders [39,113,114]. In the immune and vascular systems, DM can result in low-grade and acute inflammation [19,34,115,116,117], endothelial dysfunction [63,118,119], cardiovascular disease [7,24,25,30,31,120,121,122,123,124,125,126,127], and impairment of the neurovascular unit [3,22,41,94,97,101,119,128,129,130].

## 4. New Treatment Strategies to Address Cellular Metabolism Dysfunction

Metabolic disorders represent a significant consideration for the treatment of neurodegenerative disease. In some cases, if the disorder is recognized early in the course of the disease, neurodegenerative disability may be limited. Effective and timely treatment may inhibit both the progression of DM and diseases of the nervous system, such as cognitive loss [26,28,33,52,69,70,84,94,104,120,127,131,132,133,134,135,136,137,138,139]. Although the use of current pharmaceuticals and diet modification can assist in the treatment of DM and metabolic disorders to prevent hyperglycemic events, these strategies have potential risks that can decrease organ mass, affect cellular organelles, and lead to neuronal loss through processes that involve autophagy [104,124,140,141]. In regard to limiting cognitive loss, present therapies that may overlap with cellular metabolism pathways, such as the removal of ß-amyloid (Aβ) in the brain [92,142,143] and the use of cholinesterase inhibitors [89,92,144], may provide only symptomatic relief or slow disease progression over a limited period [83,89,145,146,147,148,149,150,151,152,153]. Additional factors that influence cognition involve low education in early life, high blood pressure, vascular disease, and tobacco use [83,84,154]. More recent studies also focus on the role of increased exercise programs for individuals that can assist with metabolic disease and neurodegenerative disorders that involve dementia and PD [8,11,12,13,41,52,155,156,157,158,159,160]. Progressive vascular disease during metabolic disorders also can lead to memory impairment and cognitive loss [55,151,155,161,162,163,164,165]. Given these challenges to overcome cognitive loss in the setting of metabolic disorders, new pathways of discovery are necessary to target novel pathways for disease onset and treatment. Innovative avenues that can address these requirements rely upon our understanding of metabolic homeostasis, programmed cell death pathways, the mechanistic target of rapamycin (mTOR) and its associated pathways of mTOR Complex 1 (mTORC1) and mTOR Complex 2 (mTORC2), AMP activated protein kinase (AMPK), growth factor signaling with erythropoietin (EPO), and underlying risk factors such as the apolipoprotein E (APOE-ε4) gene (Table 1).

## 5. Cellular Metabolism and the Role of Apoptosis, Autophagy, and Pyroptosis

Programmed cell death pathways during metabolic disorders can affect neuronal cell integrity and lead to the onset of multiple neurodegenerative disorders in the nervous system [94,128,130,149,166,167,168,169,170,171] (Figure 1). Programmed cell death, a biological process that can represent cellular suicide, can be a significant factor that can oversee the activation of inflammatory pathways during metabolic disorders, such as DM [7,19,39,104,119,126,127,135,172,173,174]. Included in these pathways of programmed cell death are apoptosis, autophagy, and pyroptosis [175,176,177,178,179,180]. 

In regard to apoptosis, this “cell-suicide” pathway has both an early and late phase [85]. The early phase consists of phosphatidylserine (PS) membrane asymmetry loss on the plasma membrane [181,182,183,184,185]. The later apoptotic phase results in deoxyribonucleic acid (DNA) degradation in the genome [186,187,188,189,190,191,192]. Loss of membrane PS asymmetry can lead inflammatory cells, such as microglia, to target, engulf, and remove injured cells [111,181,193,194]. This process is reversible if the engulfment of inflammatory cells can be stopped to allow the remaining functional cells expressing membrane PS residues to be rescued [38,195,196,197]. Apoptotic cell death is mediated through a cascade activation of nucleases and proteases that involve caspases [66,186,187,198,199,200,201,202,203]. As a result, once the destruction of cellular DNA occurs, this process may not be reversible [145]. The modulation of apoptotic pathways can minimize cognitive loss during acute insults [178,191,198,204,205,206]. Fostering anti-inflammatory pathways can improve cognitive performance and limit cellular apoptosis [91,200]. 

Autophagy processes can recycle cytoplasmic organelles and components for eventual tissue remodeling [37,69,86,87,175,177,207,208,209,210,211,212,213,214,215,216]. There are subtypes of autophagy processing. Macroautophagy recycles organelles and sequesters cytoplasmic proteins and organelles into autophagosomes that combine with lysosomes for degradation and recycling [87,217,218]. Microautophagy refers to the process of invagination of lysosomal membranes for the sequestration and digestion of cytoplasmic components [219]. Chaperone-mediated autophagy relies upon cytosolic chaperones to transport cytoplasmic components across lysosomal membranes [220,221,222]. 

Interestingly, the autophagy–lysosome pathway plays an important role in inflammatory injury during infections, such as with the pathogen severe acute respiratory syndrome coronavirus (SARS-CoV-2) [223,224,225,226,227,228,229,230,231]. Yet, processes involving autophagy hold a significant role in cognitive function [27,95,98,232,233,234,235]. The activation of autophagy can reduce tau and Aß neurotoxicity [236,237,238]. Autophagy can target Aß levels in the brain as one possible component to limit memory loss [98,146,236,239]. 

In relation to pyroptosis, this form of programmed cell death can affect inflammatory pathways in the nervous system during metabolic disease [3,39,145,191,240,241,242,243]. Pyroptosis begins with the generation of a supramolecular complex, termed the pyroptosome or the inflammasome, that can promote caspase activation to include caspase 1, caspase 4, and caspase 5. In addition, pyroptosis employs permeabilization of the plasma membrane through gasdermin protein family members. Gasdermin proteins contain both an N-terminal domain with intrinsic pore-forming properties and a C-terminal domain that can block the pore-forming properties of the N-terminal domain. Disruption of the linker sequence that binds the N-terminal and the C-terminal domains permits the N-terminal domain fragment to generate pores in the plasma. Cellular membranes are then able to release pro-inflammatory cytokines such as interleukin-1 family members. Inflammatory factors can control a balance in regard to assisting or hampering cell survival. This can be controlled by mechanisms that require gasdermin. For example, interleukin-1 family members lack a signal plasma membrane peptide that would normally allow their cellular release and therefore require gasdermin proteins to generate membrane pores [47,244]. This opening of cell membrane pores can result in the rupture of cell membranes, the release of cytokines, and other damage-associated molecular pattern (DAMP) molecules, which includes DNA and adenosine triphosphate (ATP). DAMPs can lead to the activation of the NLR family pyrin domain containing 3 (NLRP3) inflammasome in the canonical inflammasome pathways. The noncanonical inflammasome pathway is generated by binding of lipopolysaccharide proteins that can be found on Gram-negative bacteria leading to caspase 4 and caspase 5 activation. As a result, pyroptosis, in addition to apoptosis and necroptosis, may lead to pro-inflammatory responses that can cause cytokine storm and cell death [241]. These factors also play a role during DM and diabetic wound healing. Pro-inflammatory mediators, such as the NLRP3 inflammasome, can activate caspase 1 and cytokines, result in metabolic stress, and cause cell death and poor wound healing [242]. During periods of oxidative stress and reactive oxygen species (ROS) release, pyroptosis under inflammatory and toxic environments can have a significant role in affecting cognition, AD, and DM complications, which include neuronal and vascular disease [21,41,56,86,132,204,224,245].

## 6. A Central Role for the Mechanistic Target of Rapamycin (mTOR) in Cellular Metabolism

The mechanistic target of rapamycin (mTOR) is a 289-kDa serine/threonine protein kinase that is encoded by a single gene *FRAP1* [6,81,87,212,246,247,248,249]. mTOR also is known as the mammalian target of rapamycin and the FK506-binding protein 12-rapamycin complex-associated protein 1 [222]. The target of rapamycin (TOR) was initially discovered in *Saccharomyces cerevisiae* with the genes *TOR1* and *TOR2* [249]. The agent rapamycin is a macrolide antibiotic in *Streptomyces hygroscopicus* that blocks TOR and mTOR activity [119]. 

mTOR is an integral component of the protein complexes mTOR Complex 1 (mTORC1) and mTOR Complex 2 (mTORC2) (Figure 1). mTORC1 contains Raptor, Deptor (DEP domain-containing mTOR interacting protein), the proline-rich Akt substrate 40 kDa (PRAS40), and mammalian lethal with Sec13 protein 8, termed mLST8 (mLST8) [6,81]. mTOR can control Raptor activity, which can be blocked by rapamycin. Rapamycin may block the activity of mTORC1 by binding to immunophilin FK-506-binding protein 12 (FKBP12) that normally attaches to the FKBP12 -rapamycin-binding domain (FRB) at the carboxy (C)-terminal of mTOR and blocks the FRB domain of mTORC1 [85]. However, the mechanism of how rapamycin blocks mTORC1 activity with the interaction of the domain of FRB is unclear. One consideration may involve allosteric changes in the catalytic domain as well as the inhibition of phosphorylation of protein kinase B (Akt) and p70 ribosomal S6 kinase (p70S6K) [250]. mTORC1 appears to be more sensitive to inhibition by rapamycin than mTORC2, but chronic administration of rapamycin can inhibit mTORC2 activity as a result of the disruption of the assembly of mTORC2. Deptor, also an inhibitor, blocks mTORC1 activity by binding to the FAT domain (FKBP12 -rapamycin-associated protein (FRAP), ataxia-telangiectasia (ATM), and the transactivation/transformation domain-associated protein) of mTOR. PRAS40 blocks mTORC1 activity by limiting the association of p70 ribosomal S6 kinase (p70S6K) and the eukaryotic initiation factor 4E (eIF4E)-binding protein 1 (4EBP1) with Raptor [6,85,127,246]. Akt is important in this pathway as a checkpoint since mTORC1 activity is increased once the phosphorylation of PRAS40 occurs by Akt [66,190,251,252]. This releases the binding of PRAS40 and Raptor to localize PRAS40 in the cell cytoplasm with the docking protein 14-3-3 [253,254,255]. mLST8 can promote the activity of mTOR [85]. This activation involves the binding of p70S6K and 4EBP1 to Raptor [256]. 

In relation to metabolic disease, mLST8 controls insulin signaling through the transcription factor FoxO3 [115,257], is necessary for Akt and protein kinase C-α (PKCα) phosphorylation, and is required for Rictor to associate with mTOR [257]. In addition, mTORC1 is associated with metabolic disorders [26,113,224,258] and dementia [27,39,163,259]. mTORC1 can promote lipogenesis and fat storage [260], improve glucose homeostasis [261], and may increase pancreatic ß-cell mass [262]. 

mTORC2 has differences from mTOC1 and is composed of Rictor, Deptor, the mammalian stress-activated protein kinase interacting protein (mSIN1), mLST8, and the protein observed with Rictor-1 (Protor-1) [6,85,208,263,264]. mTORC2 oversees cytoskeleton remodeling through PKCα and cell migration through the Rac guanine nucleotide exchange factors P-Rex1 and P-Rex2 and through Rho signaling. mTORC2 can foster the activity of protein kinases, including glucocorticoid induced protein kinase 1 (SGK1), a member of the protein kinase A/protein kinase G/protein kinase C (AGC) family of protein kinases. Protor-1, a Rictor-binding subunit of mTORC2, can result in SGK1 activity [265,266]. mSin1 is necessary for the assembly of mTORC2 and for mTORC2 to phosphorylate Akt [267]. Rictor and mSIN1 phosphorylate Akt at serine^473^ and promote threonine^308^ phosphorylation by phosphoinositide-dependent kinase 1 (PDK1) to increase cell survival. mTORC2 also has a role during cellular metabolism [26,47]. mTORC2 signaling is required for the maintenance of pancreatic β-cell proliferation and mass [268]. Loss of mTORC2 signaling results in insulin resistance, oxidative damage [269], and severe hyperglycemia [270].

## 7. Metabolic Cell Death and mTOR

During cellular metabolic dysfunction and neurodegenerative processes, mTOR has an intimate relationship with programmed cell death pathways (Table 1). The activation of mTOR can limit apoptotic cell death in the nervous system [271,272,273,274]. During mTOR activation, retinal ganglion cell regeneration can be fostered [275], microglia can be protected during oxidative stress exposure [276], and diabetic peripheral neuropathy can be reduced [277,278]. In addition, during the activation of mTOR, Aß toxicity can be blocked [205,237,254,279,280,281,282], vascular cell death is prevented [215,283], increased neuroplasticity can occur [284], loss of mitochondria during oxidative stress is blocked [285], neuronal differentiation can be fostered [286], neonatal central nervous system hypoxic injury is prevented [287], and reduced stroke volume with decreased apoptotic cell death dependent is promoted [198].

In some cases, the activation of autophagy with the inhibition of mTOR may be neuroprotective [37,47,61,86,87,205,207,235]. Cognitive function may be improved with low-calorie diets that promote autophagy and limit mTOR activity [288]. The activation of autophagy with decreased mTOR function may result in improved memory and more robust insulin signaling that can increase Aß clearance [289]. Additional work suggests that cognition may improve with tau clearance during autophagy activation and reduced mTOR function [238]. Autophagy activation in the setting of decreased mTOR activity also can prevent mitochondrial dysfunction [290], limit injury to dopamine-dependent cells [291], decrease reactive oxygen species release [292], and modulate neuroprotection with glutamine-dependent mechanisms [293]. During DM, mTOR inhibition can offer protection such as during cerebral ischemia [294] and is necessary for maintaining a balance between pancreatic β-cell proliferation and cell size [268]. Additional studies indicate that the dysregulation of autophagy as a central pathway can result in the progression of cognitive loss with AD and the induction of DM [99]. Studies also have shown that autophagy haploinsufficiency with deletion of the *Atg7* gene in mouse models of obesity leads to increased insulin resistance with elevated lipids and inflammation [295]. A loss of autophagic proteins Atg7, Atg5, and LC3 also can be responsible for diabetic nephropathy [296]. Autophagy offers protection by removing misfolded proteins and eliminating non-functioning mitochondria to maintain β-cell function and prevent the onset of DM [297]. Exercise in mice also has been shown to foster autophagy and regulate glucose homeostasis [298], possibly through improved insulin sensitivity [299] and supporting microglial function during acute glucose fluctuations [95].

It is important to note though that the activity of mTOR may be required to some degree, since studies suggest that dysfunction in mTOR signaling can result in cognitive impairment and synaptic dysfunction [64,85,87,235,300,301]. Autophagy activation can injure endothelial progenitor cells, lead to mitochondrial oxidative stress, and prevent new blood vessel formation during elevated glucose exposure [302]. At times, autophagy can be a component that leads to neuronal cell death [239]. Increased activity of autophagy can result in a significant loss of cardiac and liver tissue in diabetic rats during attempts to achieve glycemic control through diet modification [140]. During periods of elevated glucose, advanced glycation end products (AGEs), agents that can result in complications during DM, have been shown to lead to the induction of autophagy and vascular smooth muscle proliferation that can result in atherosclerosis [303] as well as cardiomyopathy [304]. Autophagy during elevated glucose exposure can lead to mitochondrial oxidative stress [305], impair endothelial progenitor cells, and block angiogenesis [302]. Growth factors that promote mTOR activation and limit autophagy can offer neuronal and vascular protection in the nervous system, such as during application with EPO [186,252,306]. EPO can modulate several mTOR pathways, such as PRAS40 and Akt, to lead to increased neuronal survival [253,307,308,309]. The activity of mTOR also is necessary for interneuron progenitor growth in the brain during autophagy inhibition [310]. 

In addition to its role in the nervous system, mTOR is necessary for the proper function of several metabolic pathways [6,26,27,37,113,117,127,130,226,311,312]. mTOR activation with the pathways of p70S6K and 4EBP1 can promote insulin secretion in pancreatic β-cells and increase resistance to β-cell streptozotocin toxicity and obesity in mice [262]. In contrast, the loss of p70S6K activity results in hypo-insulinemia, insulin insensitivity to glucose secretion, glucose intolerance, and decreased pancreatic β-cell size [313]. mTOR activation in patients with metabolic syndrome has also been found to be diminished and possibly responsible for insulin resistance with an increased risk of vascular thrombosis [314]. The activation of mTOR pathways has been tied to pancreatic β- cell protection against cholesterol-induced apoptosis [315], increased neuronal cell survival in cell models of DM [316], and decreased glucolipotoxicity [317]. Yet, mTOR inhibition leads to reduced β-cell function, insulin resistance, and limited insulin secretion associated with the progression of DM [318]. Loss of mTOR activity can also increase mortality in a mouse model of DM [319]. Furthermore, the translocation of glucose transporters to the plasma membrane in skeletal muscle is impacted during mTOR inhibition [320]. 

mTOR can, under some scenarios, control cellular metabolism to preserve cognition. mTOR activity can oversee insulin signaling in experimental models of AD and maintain astrocyte viability [321], prevent endothelial cell dysfunction during periods of hyperglycemia [322], and maintain glucose homeostasis [261]. mTOR may offer additional protection as a component of the Mediterranean diet and nutrition. mTOR may reduce Aβ toxicity in astrocytes via enhanced Akt activity through the consumption of polyphenols in olives and olive oil, which may be linked to the onset of AD [321].

## 8. Metabolic Cell Death and AMP Activated Protein Kinase (AMPK)

The AMP activated protein kinase (AMPK) plays an important role in both metabolic and neurodegenerative disorders as well as in other disease entities that involve inflammation and infection [8,26,86,177,264,323,324,325] (Figure 1). AMPK controls mTORC1 activity through the hamartin (tuberous sclerosis 1)/tuberin (tuberous sclerosis 2) (TSC1/TSC2) complex that inhibits mTORC1 function [325,326]. Modulation of the TSC1/TSC2 complex also can be overseen though phosphoinositide 3-kinase (PI 3-K), Akt, and its phosphorylation of TSC2. Extracellular signal-regulated kinases (ERKs), protein p90 ribosomal S6 kinase 1 (RSK1), and glycogen synthase kinase -3β (GSK-3β) can oversee the activity of the TSC1/TSC2 complex as well. TSC2 functions as a GTPase-activating protein (GAP) that changes G protein Rheb (Rheb-GTP) into the inactive GDP-bound form (Rheb-GDP). During Rheb-GTP activation, Rheb-GTP associates with Raptor to oversee the binding of 4EBP1 to mTORC1 and increase mTORC1 activity [327]. AMPK phosphorylates TSC2 to increase GAP activity to change Rheb-GTP into the inactive Rheb-GDP and to block mTORC1 activity [328]. 

As a pathway of mTOR, AMPK is a critical modulator of mTOR pathways and can oversee cellular metabolism and the pathways of programmed cell death. AMPK has been shown to affect insulin resistance and mitochondrial homeostasis [23,39,329,330]. During dietary restrictions that may increase lifespan [331], AMPK can alter cellular metabolism to shift to protective oxidative metabolism [332]. In relation to stem cell maintenance, AMPK can be necessary for resistance to senescence in mesenchymal stem cells [333] and can protect endothelial progenitor cells during periods of hyperglycemia [334]. Anti-senescence cell activity can be promoted through mTOR inhibition, AMPK activation, and the acceleration of autophagic flux [335]. In regard to neurodegeneration, AMPK activation can improve memory retention in models of AD and DM [336], assist with pathways for healthy aging [58], facilitate clearance of Aß [337] and tau [238] in the brain, diminish Aß neurotoxicity [338], and control chronic inflammation in neurodegenerative disorders [61,81,326].

AMPK employs autophagy activation to control cell survival and regulate metabolic homeostasis [6,23,41,47,113,322]. Modulation of AMPK activity may be required to increase basal autophagy activity [175,339] and maintain endothelial cell survival [322,340] during hyperglycemia. AMPK can control both apoptosis and autophagy during coronary artery disease [341] and oxidative stress cell injury [342,343]. In regard to growth factor protection in the nervous system, EPO can increase neuronal survival through increased AMPK activity and enhanced autophagy activity [344]. EPO oversees AMPK and mTOR activities to protect cells under conditions of oxidative stress [281] and inflammation [306,345,346]. The duration and concentration of EPO exposure can influence a specific level of AMPK and mTOR activity to alleviate the detrimental effects of oxidative stress [253,347]. This fine control over mTOR is important since high concentrations of EPO may result in cell death and reduce the activity of mTOR [348]. 

Interestingly, AMPK pathways are used by current agents to treat DM, such as metformin and biguanides, to limit neurodegenerative disease, such as peripheral neuropathy, demyelinating disease, and cognitive loss [27,224,329,349,350]. Metformin inhibits mTOR activity, promotes autophagy, and may function at times in an AMPK-independent manner [351]. Metformin can limit lipid peroxidation in the brain and spinal cord and decrease caspase activity to enhance cell survival [352]. Recent work also suggests that metformin through AMPK pathways can accelerate myelin recovery in animal models of multiple sclerosis [349]. In relation to infections of the nervous system that can lead to neurodegeneration, metformin reduced disability in obese patients or individuals with DM during coronavirus disease 2019 (COVID-19) [14,353].

## 9. Cellular Metabolism and the ε4 Allele of the Apolipoprotein E (APOE-ε4)

Since the sporadic form of AD represents most of the clinical cases, it can impact at least ten percent of the global population over the age of 65 [6,47,73,86,87,91,92,93]. Although multiple factors and cellular pathways may precipitate AD, individuals with the ε4 allele of the apolipoprotein E (APOE-ε4) gene have an increased risk of late-onset AD [215,237,354,355,356] (Figure 1). Apolipoprotein E (APOE) is produced in the liver and is important for cellular metabolism by regulating lipid homeostasis and the transport of cholesterol, triglycerides, and phospholipids in the body [96,147]. In the brain, APOE is generated in astrocytes and is used through APOE receptors to transfer cholesterol to neurons [99,215]. In some cases, APOE can assist with the destruction of Aβ in the brain (Table 1). However, the isoform APOE-ε4 is not effective in the destruction of Aβ, which may result in heightened risk of the development of AD [99,237,354,357,358,359]. Individuals with two ε4 alleles may have almost 20 times the risk of developing AD. Interestingly, PS membrane exposure [360,361,362,363], an initial phase of which is apoptotic cell death, may be related to Aβ aggregation and some isoforms of APOE may prevent the aggregation of Aβ through PS membrane exposure, but this is not the case for the isoform APOE-ε4 [358].

APOE-ε4 has been shown to affect mTOR signaling and increase mTOR activity [364,365]. The ability of APOE-ε4 to alter mTOR activity as well as autophagy flux has been suggested to increase the risk for the development of cerebrovascular disease and AD due to possible deficits in synaptic plasticity [366]. In addition, studies have suggested that at least twenty-two viral diseases have been identified to cause increased risk of neurodegenerative disorders, with the most prominent presenting disease being dementia [367]. In particular, APOE-ε4 may promote the susceptibility of viral infection and cerebrovascular disease during COVID-19 [368]. The β-coronavirus family virion, severe acute respiratory syndrome (SARS) -CoV-2 (SARS-CoV-2), has led to multiple and repeated infections throughout the global population [8,27,223,224,225,226,227,228,312,350,369,370,371,372]. Coronaviruses are ribonucleic acid (RNA) viruses and are members of the family of *Coronaviridae* and the subfamily of *Orthocoronavirinae* [27,241,373,374,375,376,377]. SARS-CoV-2 can attach to host cells, such as in the nasal epithelial region [378] and the brain [117], and lead to a heightened response of the immune system [10,375,379,380,381,382].

Cognitive disability can ensue after infection with SARS-CoV-2 [27,224,311,377,383,384]. The cognitive loss can be part of a long-COVID syndrome [227]. Long-COVID, also known as long-haul COVID, post-acute COVID-19, and chronic COVID, represents long-term effects that can occur following acute SARS-CoV-2 infection. Multiple mechanisms can account for this chronic syndrome, including metabolic pathways, apoptosis, autophagy, oxidative stress, mitochondrial dysfunction, and cytokine release [56,73,74,86,87,88,91,177,178,180,206,212,251,385,386,387,388,389,390,391,392,393]. With the knowledge of the role of APOE-ε4 in metabolism and autophagy signaling, APOE-ε4 has been linked to the effects of long COVID and dementia. Individuals with two ε4 alleles of APOE-ε4 have reduced expression of antiviral defense genes and have more severe neuroinflammation and microvascular injury in the brain [151,355,394]. APOE-ε4 during SARS-CoV-2 infection and long COVID may result in cognitive loss and cerebrovascular disease in the nervous system [11,30,61,66,85,167,177,199,395,396].

## 10. Future Perspectives

Metabolic disease, which includes DM, represents a significant healthcare concern for the global population (Table 1). The prevalence of DM continues to increase and by the year 2045 seven hundred million individuals are expected to suffer from DM according to the International Diabetes Federation [1]. Of further concern, it is estimated that approximately seven million individuals over the age of eighteen are undiagnosed with DM. Although the use of current pharmaceuticals and diet modification can assist in the treatment of DM and metabolic disorders, these treatments cannot reverse disease progression and have potential risks that can decrease organ mass, affect cellular organelles, and lead to neuronal loss through processes that involve autophagy. Of additional concern is the role of metabolic disorders in fostering neurodegeneration, especially since DM can be a critical factor for the development of cognitive loss and dementia. Coupled to these concerns for the onset of neurodegenerative disease as a result of underlying metabolic disorders is the limited treatment arsenal for disorders such as cognitive loss, now considered to be the seventh leading cause of death. Overcoming these challenges requires the implementation of novel and innovative strategies. The focus upon metabolic homeostasis, programmed cell death pathways, mTOR and its associated pathways of mTORC1, mTORC2, AMPK, growth factor signaling with EPO, and underlying risk factors such as the APOE-ε4 gene can offer new directions to identify potential therapeutic strategies for metabolic disorders and neurodegenerative disease (Figure 1).

The examination of these pathways, each intimately linked to one another, offers a number of valuable attributes. For example, mTOR activation can lead to retinal ganglion cell regeneration, a reduction in diabetic peripheral neuropathy, and limit Aß toxicity in the nervous system. Trophic factors, such as EPO, can offer cellular protection against neurotoxic insults by also fostering the activation of mTOR. Components of the mTOR pathway, such as mLST8, can oversee insulin signaling to help maintain glucose homeostasis. mTORC1 can promote lipogenesis and fat storage, improve glucose homeostasis, and increase pancreatic ß-cell mass, and mTORC2 signaling is necessary for the maintenance of pancreatic β-cell proliferation and mass. However, under some conditions, the activation of autophagy with the inhibition of mTOR may be necessary to prevent neurodegeneration. As an example, the activation of autophagy with blocked mTOR function may result in improved memory and more robust insulin signaling that can increase Aß clearance in the nervous system. The dysregulation of autophagy also may result in the progression of cognitive loss with AD and DM. AMPK activation during the inhibition of mTOR leads to memory retention, limits lipid accumulation and obesity, and results in neuroprotection. However, under alternate cellular conditions, AMPK inhibition with active mTOR signaling is necessary to offer protection to pancreatic islet cells, limit Aβ toxicity, and block nervous system inflammation.

## 11. Conclusions

A fine balance between glucose homeostasis, programmed cell death pathways, and mTOR signaling pathways is required. As a result, future studies are warranted to pursue such directions. For example, biological feedback pathways with mTOR, such as through AMPK inhibition, are believed to exist to prevent excessive mTOR activity. Periods of elevated mTOR activity can be detrimental and result in glucose intolerance by inhibiting the insulin receptor substrate 1 (IRS-1) [397]. In addition, the presence of growth factors can affect this balance, since the duration and concentration of EPO exposure can affect AMPK and mTOR activity to offer cellular protection with controlled EPO concentrations but limit the activity of mTOR and lead to cell death during elevated EPO concentrations.

Additional investigations must also seek to understand the underlying risk factors for neurodegenerative disease that can occur as a result of metabolic disorders. The role of APOE-ε4 as a significant risk factor for developing AD and the function of APOE-ε4 to regulate lipid homeostasis and the transport of cholesterol, triglycerides, and phospholipids in the body offers critical support for this conjecture. Aβ aggregation in the brain appears to be linked to the inability of APOE-ε4 to oversee apoptotic signaling with PS membrane exposure. Furthermore, APOE-ε4 has been shown to affect mTOR signaling, increase mTOR activity, and alter autophagy flux that can increase the risk for the development of cerebrovascular disease and AD. In addition, APOE-ε4 also may increase the susceptibility of viral infection and cerebrovascular disease during COVID-19 as well as promote long-term disability with dementia and long-COVID syndrome. Individuals with two ε4 alleles of APOE-ε4 have limited expression of antiviral defense genes and, as a result, have more severe neuroinflammation and microvascular injury in the brain. The future offers new challenges to unravel the complex and critical pathways that tie neurodegenerative disorders to metabolic disease and develop new therapeutic avenues for these diseases that impact the global population.

## Figures and Tables

**Table 1:** Highlighting Cellular Metabolism: A Fundamental Component of Degeneration in the Nervous System.

The prevalence of cellular metabolic dysfunction disorders, such as diabetes mellitus (DM), has increased from nine and one-half percent during the period of 1999 to 2002 to twelve percent during the period of 2013 to 2016.
Metabolic disorders affect all systems of the body and especially can involve the central nervous system and the peripheral nervous system. As a result, cellular metabolic dysfunction can be a significant contributor to cognitive loss and dementia.
Strategies that can address cellular metabolism, apoptosis, autophagy, and pyroptosis, the mechanistic target of rapamycin (mTOR), AMP activated protein kinase (AMPK), growth factor signaling with erythropoietin (EPO), and risk factors such as the apolipoprotein E (APOE-ε4) gene and coronavirus disease 2019 (COVID-19) provide fruitful directions for the treatment of metabolic disorders coupled to neurodegenerative disease.
Metabolic pathways such as AMPK can oversee programmed cell death pathways during vascular disease, oxidative stress cell injury, and growth factor protection in the nervous system. In addition, pyroptosis may lead to pro-inflammatory responses that can cause cytokine storm and cell death that require additional oversight by AMPK during chronic inflammation.
Conditions such as long-COVID syndrome resulting from multiple mechanisms can account for this chronic syndrome that involves metabolic pathways, apoptosis, autophagy, oxidative stress, mitochondrial dysfunction, and cytokine release as well as APOE-ε4.
A fine balance between glucose homeostasis, apoptosis, autophagy, pyroptosis, and mTOR signaling with AMPK is required since, at times, the activation of autophagy with blocked mTOR function may result in improved memory, but in other scenarios the dysregulation of autophagy may lead to the progression of cognitive loss during cellular metabolic dysfunction.

## Data Availability

Not applicable.
